# A qualitative study of stakeholder views on the use of a digital app for supported self-management in early intervention services for psychosis

**DOI:** 10.1186/s12888-021-03317-9

**Published:** 2021-06-19

**Authors:** Thomas Steare, Maria Giorgalli, Katherine Free, Jasmine Harju-Seppänen, Syeda Akther, Michelle Eskinazi, Puffin O’Hanlon, Helen Rostill, Sarah Amani, Brynmor Lloyd-Evans, David Osborn, Sonia Johnson

**Affiliations:** 1 Division of Psychiatry, University College London, Maple House, London, W1T 7NF UK; 2 Research Department of Clinical, Educational and Health Psychology, University College London, London, UK; 3 Oxford Health NHS Foundation Trust, Oxford, UK; 4 Oxford Institute of Clinical Psychology Training and Research, Warneford Hospital, University of Oxford, Oxford, UK; 5 R&D Department, Camden and Islington NHS Foundation Trust, London, UK; 6 University of Surrey, Guildford, UK; 7 Surrey and Borders Partnership NHS Foundation Trust, Leatherhead, Surrey, UK; 8 EIP Programme (South of England), NHS England, Oxford, Oxfordshire UK

**Keywords:** Psychosis, Self-management, Smartphone, Feasibility study, Patient experience

## Abstract

**Background:**

Digital tools such as Smartphones have the potential to increase access to mental health support including self-management interventions for individuals with psychosis, and ultimately to improve outcomes. Self-management strategies, including relapse prevention and crisis planning and setting personal recovery goals, are intended to assist people with long-term conditions to take an active role in their recovery, with evidence for a range of benefits. However, their implementation is inconsistent, and access and uptake need to be improved. The current study explores the acceptability of a Smartphone app (My Journey 3) that has been developed to facilitate supported self-management in Early Intervention in Psychosis (EIP) services.

**Methods:**

Semi-structured one-to-one interviews were conducted with twenty-one EIP service users who had access to My Journey 3 as part of a feasibility trial, and with thirteen EIP service clinicians who were supporting service users with the app. Interviews focused on the acceptability and usability of My Journey 3. Data was coded to themes based on the Acceptability of Healthcare Interventions framework.

**Results:**

Many service user participants found My Journey 3 to be acceptable. The symptom and medication trackers in particular were described as helpful. A smaller number of service users disliked the intervention. Individual-level factors that appeared to influence acceptability and engagement included recovery stage and symptom severity. Clinicians tended to report that My Journey 3 was a potentially positive addition to service users’ care, but they often felt unable to provide support due to competing demands in their work, which in turn may have impacted acceptability and usage of the app.

**Conclusions:**

Our findings suggest that the app is perceived as having potential to improve users’ capacity to self-manage and work towards recovery goals, but barriers prevented many clinicians providing consistent and effective support as intended. Further evaluation of supported self-management apps in psychosis is warranted but needs to address implementation challenges from the start.

## Background

Digital technology is increasingly advocated as a means to enhance care and overcome barriers to treatment access within healthcare services. The recent National Health Service (NHS) Five Year Forward View for Mental Health emphasises the importance of developing and implementing digital technologies for the development and improvement of mental health services, with a specific emphasis on new digital applications (apps) [1]. Smartphones, which are widespread and easily accessible, are a potentially viable mechanism to support the delivery of the care of first-episode psychosis [2]. People with psychosis commonly own Smartphones, use them in a similar way to the general population [3–5] and are willing and able to use digital health interventions (DHIs) [6, 7].

Supported self-management is intended to assist people with long-term health conditions in taking an active role in their recovery. Although there is not a universal definition, key components of supported self-management interventions consist of psycho-education around mental illness and treatment; creating a personal plan to aid prevention of relapse; encouraging medication adherence; identifying and planning personal recovery goals; and developing coping strategies [8]. In recent meta-analyses, self-management interventions were found to improve a range of outcomes for adults with severe mental illness [9], and reduce the risk of future relapse for those with schizophrenia [10].

Despite the promise of self-management approaches there is a lack of well-evaluated tools to support delivery in psychosis. Smartphones have the potential to overcome implementation barriers and help embed the delivery of supported self-management within Early Intervention in Psychosis (EIP) Services (community mental health teams that provide support for individuals following a first-episode of psychosis). A number of Smartphone apps that deliver self-management have been developed by researchers, and have been found to be feasible, acceptable and potentially clinically beneficial for adults with psychosis [11–13]. A recent systematic review of DHIs for schizophrenia and other psychotic disorders found that symptom monitoring and management was the most frequent goal of DHIs in schizophrenia, with supported self-management not explicitly identified as a goal, although several “general purpose” apps may have had goals relevant to this [14]. The overall quality of evidence was noted to be low in this field, despite substantial activity, with few studies yielding clear evidence on effectiveness.

To our knowledge, three studies have published trial results of supported self-management Smartphone apps delivered within EIP services. In our feasibility trial (the ARIES study), we were able to deliver a Smartphone app, developed with substantial stakeholder input, within EIP services. The app, known as My Journey 3, was designed to increase the implementation of supported self-management for adults with first-episode psychosis [15]. Recruitment and retention in the feasibility trial were largely successful, but we were concerned that usage rates of the app varied greatly between participants and tended to be relatively low. Prior to further investigation of the clinical effectiveness of My Journey 3, we identified a need to focus further on strategies for engaging service users and clinicians with its use.

In a proof-of-concept trial led by Bucci and colleagues, a cognitive-behavioural therapy-informed self-management app (Actissist) was shown to confer benefits over a passive control app [12]. Finally, in an open randomised controlled trial (RCT), an app which was integrated into electronic health records of participating NHS Trusts and aimed to encourage self-management and symptom monitoring was associated with improvement in psychotic symptoms in participants with recent-onset psychosis [16]. Trials of other promising self-management apps for psychosis within mental health services are ongoing, including a trial of the EMPOWER app which features an algorithm monitoring users’ early warning signs in addition to access to self-management interventions [17].

A key impediment to realising potential benefits from DHIs in psychosis and other mental health conditions is generally low rates of real-world usage. In order to ensure that DHIs are meaningful, acceptable and have high user engagement, a better understanding of the factors driving acceptability, implementation, and initial and continuing engagement is needed, as well as an understanding of service users’ and healthcare professionals’ experience of using relevant apps over time [3, 18–21]. Therefore, we aimed to explore the acceptability of My Journey 3 through interviews with both clinical providers and service users whom participated in the ARIES study, Our findings were intended to aid our understanding of the feasibility and appropriateness of proceeding to a full RCT of this technology, and to inform the development, delivery and implementation of the app and similar digital self-management tools for psychosis.

## Methods

### Setting

This qualitative study was nested within the ARIES study, a feasibility RCT the feasibility, acceptability, and development of a supported self-management Smartphone app for EIP services. The protocol and main outcomes of the feasibility trial have already been reported [15, 22]. The study recruited 40 participants who were randomised to receive either My Journey 3 as an addition to their clinical care or treatment as usual with no enhancement. The setting for the study was six EIP services in three NHS Foundation Trust catchment areas in England. The participating areas included inner city and more rural areas and are ethnically and social diverse. The aim of the nested qualitative study was to understand the acceptability of My Journey 3 from both service users’ and clinicians’ perspectives.

### Participants

Participants in this qualitative study were involved in the ARIES feasibility trial, either as service user participants or as clinicians. Service user participants were eligible for the ARIES study if they met the following criteria: 1) aged ≥16 years, 2) on the caseload of one of the participating EIP services, 3) owned a Smartphone with an Android operating system (we lacked the resources to implement My Journey 3 on multiple platforms), 4) had capacity to provide informed consent, 5) were able to communicate in English, and 6) were not assessed by the EIP service as posing a high risk to researchers even on NHS premises. Recruitment to the ARIES study was conducted over a 7-month period in 2017. Service user participants were eligible for the nested qualitative study if they were randomised to the My Journey 3 treatment group within the ARIES study. Clinicians who supported service user participants with My Journey 3 as part of the ARIES study were eligible for the qualitative study.

### Intervention

My Journey 3 has been developed to digitally deliver adapted paper-and-pen self-management tools used in NHS services, ensuring that these are effectively initiated and sustained in a format that is accessible and convenient for service users, carers and clinicians [23, 24]. In designing My Journey 3 we collaborated with researchers, digital health experts, EIP service users and clinicians. Users of the app can create and store a relapse prevention plan with the aid of an EIP service clinician. The relapse prevention plan section allows users to identify triggers, early warning signs and personalised coping strategies and to also create a plan to implement in the case of a mental health crisis. My Journey 3 features a symptom tracker which enables users to monitor symptoms and early warning signs, and a section where users can record recovery plans and identify steps to achieving them. Users have access to information on mental health, medication, and mental health services in a psychoeducation section of the app including short videos Medication adherence can be tracked via a ‘Pill Tracker’, which features a daily reminder to encourage users to enter if they have taken their psychiatric medication.

My Journey 3 is suitable for independent use once service users are familiar with it but is primarily intended for use as a supported self-management tool in collaboration with EIP service clinicians who can assist with the completion of the self-management components and initial set-up and should then review progress along with service users. Within the ARIES study, participants allocated to the intervention arm attended an app training session with a supporting EIP service clinician and the study researcher. At the meeting they were introduced to My Journey 3, downloaded it on to their Smartphones and were shown how to navigate and use the app. Participants were encouraged to input information to specific components of My Journey 3 including initial personal recovery plans, relapse prevention plans, and crisis plans, in collaboration with the supporting EIP service clinician.

Supporting EIP service clinicians were asked by the researcher to provide regular encouragement to use it during the study period, and to further discuss and develop discuss participants’ personal recovery goals and relapse prevention plans at future clinical appointments. Clinicians’ understanding of My Journey 3 was from the training session only, but they would usually have had some pre-existing understanding of self-management approaches from previous clinical training, although structured self-management tools such as relapse prevention plans were only sporadically used in the participating services. A limited structure was provided for continuing collaboration between clinicians and service users, with clinician support for use of the app not manualised, incentivised, or formally recorded.

### Procedures

All service user participants who received My Journey 3 were invited to take part in individual semi-structured qualitative interviews at a four-month follow-up meeting: this followed on from the quantitative outcome measures at this timepoint [15]. The researcher contacted participants and explained the procedure for the research meeting. They were informed that the meeting would feature a short optional research interview which would explore their experience of using My Journey 3 and of its acceptability.

At the research meeting, participants were provided with a study information sheet and were asked to provide written informed consent if they agreed to take part in the audio-recorded interview. The interview followed the completion of a battery of questionnaires on socio-demographic characteristics and mental health outcomes and a clinical interview administered as part of the feasibility trial. Participants were given £20 as a thank you for attending the follow-up meeting, and they were assured that there were no consequences if they declined the interview.

All clinicians who had been supporting participants with My Journey 3 were invited to take part in a separate interview. Clinicians were approached by the researcher at the same time the service user participant they had been supporting was contacted to complete the four-month follow-up meeting. The researcher introduced the qualitative study and provided the study information sheet via email. Informed consent was provided beforehand, and prior to the interview participants completed a short demographic questionnaire.

A semi-structured interview schedule was developed by the study team with questions designed to assess feasibility and acceptability of the intervention and barriers and facilitators to its use, ensuring that key topics were discussed but that new themes of concern to participants could also emerge. The interview schedules were piloted with service user and clinician participants who took part in a one-month field study designed to test the app’s usability prior to the feasibility trial. Most service users and clinicians were familiar with the interviewer from previous meetings for the ARIES study. All participants were aware beforehand that the main purpose of the interview was to establish the usability and acceptability of My Journey 3.

The service user interview schedule covered:

- The usability and acceptability of My Journey 3
- Positive and negative aspects of My Journey 3
- The reported impact of My Journey 3 on their life
- Facilitators and barriers to using My Journey 3
- Views on the clinician support with My Journey 3

The clinician interview schedule covered:

- Positive and negative aspects of My Journey 3
- The experience of supporting service users with My Journey 3
- Facilitators and barriers to incorporating use of My Journey 3 in EIP clinical management and to providing support to service users with this.

One-to-one interviews were conducted by TS, JHS & SA, whom prior to the study undertook training in qualitative interviewing. Interviews took place from August 2017 to March 2018 and were digitally recorded and transcribed verbatim. Identifiable information was removed from the transcripts to preserve anonymity. Manuscripts were imported to QSR NVivo 11 for Windows for data analysis.

### Analysis

Data were analysed using a deductively driven thematic analysis approach [25], and an established theoretical framework (the Acceptability of Healthcare Interventions framework) [19] designed to guide the assessment of acceptability of interventions from both the users’ and facilitators’ perspective. Data was accordingly coded into seven constructs relating to the acceptability of the intervention. These were: affective attitude, burden, ethicality, intervention coherence, opportunity costs, perceived effectiveness, and self-efficacy (Table 1). Data regarding suggestions on how to improve My Journey 3 content, design or delivery were coded and are reported separately. All transcripts were first read by TS, MG & KF for data familiarisation, and then independently coded. The research team met regularly during data analysis to compare codes and establish consensus and reliability. Coding began after the final interview was conducted.

**Table 1:** Codebook adapted from the Acceptability of Healthcare Interventions framework [19]

Construct	Description
Affective Attitude	How the participant feels about taking part in My Journey 3
Burden	The perceived amount of effort that is required to participate in My Journey 3
Ethicality	The extent to which My Journey 3 has a good fit with the participant’s value system
Intervention Coherence	The extent to which the participant understands My Journey 3 and how it works
Opportunity Costs	The extent to which benefits, profits or values must be given up to engage with My Journey 3
Perceived Effectiveness	The extent to which My Journey 3 is perceived as likely to achieve its purpose
Self-efficacy	Participants’ confidence that they can perform the behaviours required to use and engage with My Journey 3

### Research team

TS, JHS, SA, MG & KF were employed as Research Assistants, and all have a research-based Master’s degree and an interest in digital mental health. The conduct of the interviews and data analysis were overseen by SJ, DO & BLE who are experienced in mixed-methods health service research.

## Results

All participants who received My Journey 3 (*n* = 21) as part of the ARIES feasibility trial consented and took part in semi-structured interviews. Thirteen of the sixteen EIP service clinicians who were supporting participants with My Journey 3 participated in the qualitative study (1 declined, 2 uncontactable).

Demographic data regarding service users are displayed in Table 2. The majority of the sample had access to My Journey 3 on their own Smartphone (*n* = 19, 90%). The study team lent two participants an Android Smartphone as part of the ARIES feasibility trial due to problems with their own Smartphones. Interviews were conducted at participants’ own homes (*n* = 10), at their EIP service (*n* = 7), at University College London (*n* = 3) or via telephone (n = 1). Interviews with service users lasted from 5 to 20 min.

**Table 2:** Participant demographics

	Service users	Clinicians
	N (%)	N (%)
**Gender**		
Female	5 (23.8)	7 (53.9)
Male	16 (76.2)	5 (38.5)
Missing	0	1 (7.7)
**Age, years – mean (range)**	29.8 (18 to 52)	
**Ethnicity**^a^		
White British	9 (42.3)	8 (61.5)
Any other white/Mixed white	3 (14.3)	1 (7.7)
Black African	3 (14.3)	3 (23.1)
Black Caribbean	1 (4.8)	0
Asian Other	2 (9.5)	1 (7.7)
Other/Mixed other	3 (14.3)	0
**Highest level of education**		
Postgraduate training or qualification	1 (4.8)	
Undergraduate degree	5 (23.8)	
Some University but no degree	2 (9.5)	
HND or professional qualification	1 (4.8)	
A Levels or equivalent	2 (9.5)	
GCSEs or equivalent	5 (23.8)	
No qualifications	4 (19)	
Missing	1 (4.8)	
**Employment status**		
Employed – more than 16 h a week	5 (23.8)	
Employed – less than 16 h a week	2 (9.5)	
Voluntary work	3 (14.3)	
In study or training	1 (4.8)	
Unemployed or exempt due to disability	8 (38.1)	
Missing	2 (9.5)	
**ICD-10 diagnosis**		
F20-F29: Schizophrenia or related disorder	14 (66.7)	
F30-F39: Mood disorder	5 (23.8)	
Missing	2 (9.5)	
**Duration of access to My Journey 3, weeks – mean (range)**	6.94 (5.71 to 10.29)	
**Previously used a mental health app**		
Yes	4 (19.1)	
No	17 (80.9)	

^a^Due to rounding, percentages may not add up exactly to 100%. All statistics are reported N (%) unless otherwise specified

Characteristics of clinician participants are displayed in Table 2. Clinicians were all the care-coordinators of the participants they were supporting with My Journey 3, however job titles varied. All clinician interviews took place at the EIP service where they were based. Interviews with clinicians lasted from 9 to 26 min.

### Affective attitude

Most service user participants were happy to be using My Journey 3 alongside EIP service support and found using the app to be a positive experience.

*“It’s a really good app for everybody to have” (Service User 5, 52 years old).*

*“There’s nothing that I’ve seen anyway like this, so it’s quite bespoke and I think it could be very helpful” (Service User 7, 27 years old).*

One of the many reasons service users enjoyed engaging with My Journey 3 was that they felt safe expressing themselves, for example inputting their current symptoms and mood. It was perceived as a neutral place to share without judgement.

*“It is like a safety zone I’d say, like a safe place to actually talk” (Service User 9, 25 years old).*

Many participants felt that My Journey 3 allowed for greater control over their recovery by helping them to maintain a routine. The app also allowed for greater insight as it helped overcome barriers, such as forgetfulness, which may prevent service users from being aware of their recent progress or decline of their mental state.

*“People ask you if it’s getting better or worse and it’s very difficult to remember, so having the charts was helpful and like I don’t know stuff arranged in charts just makes you feel more in control I suppose, it’s quite satisfying” (Service User 4, 31 years old).*

In contrast, some participants were less positive about My Journey 3 and Smartphone apps in general.

*“Unfortunately, it wasn’t for me. I’m not a person that likes spending time on my phone. I’m not one that’s always using the phone. I find working with my psychologist and my CPN worker, I find it more beneficial than actually looking at an app, so for someone else it would probably be fantastic, but for a non-gadget person it’s not really my cup of tea.” (Service user 2, 51 years old).*

The majority of care coordinators expressed a positive attitude towards My Journey 3 as a healthcare tool. One benefit identified was that it allows service users to easily access support outside of the care from the early intervention team and health care professionals.

*“I think they’ll find it very very beneficial, ‘cos we’re not there most of the time, you know this guy’s there by himself and we’re just there to support them, monitor their mental state …” (Clinician 1).*

### Perceived effectiveness

A large number of service user participants said they found My Journey 3 helpful. Many were unsure whether changes in their mental health and/or lifestyle since using My Journey 3 were due to the app. However, a few participants believed it had a recognisable impact on them.

*“It’s made an effect you know for activities and relationships, I think it’s done like a real boost on activities and my relationship with friends and family.” (Service User 9, 25 years old).*

Positive changes in service users’ lifestyles and routines were attributed to certain features of My Journey 3. In particular many service users described symptom tracker as an important and helpful part of the app, facilitating a feeling of control and personal ownership of their mental health.

*“I used the mood [symptom] tracker more than I did the relapse prevention and it made me think about what I was thinking, feeling, seeing, hearing before something bad happens … it’s just gets me involved and that’s what I need to stay positive” (Service User 19, 22 years old)*

My Journey 3 was seen as being effective in improving adherence to medication, with many participants saying that the Pill Tracker was one of the most helpful aspects of the app. Several service users stated that prior to using My Journey 3 they often forgot to take medication or that they would sometimes mistakenly take the wrong dosage.

*“I’ve used the pill tracker to start off with, as an alarm to remind me of the medication which I thought was very helpful, because before I had the app I’ve taken my medication twice quite a lot, so yeah that’s really helpful.” (Service User 9, 25 years old)*

The perceived effectiveness of My Journey 3 seemed to be influenced by the service user’s stage of recovery in psychosis and whether they felt the intervention was relevant to them. Some service users felt My Journey 3 was not relevant to them as they were already in an advanced stage of their recovery.

*“ … ‘cos obviously I’m in a place where I’m like recovered, so I didn’t feel like it was that essential to be like tracking my mood and doing these things and like setting these goals and you know listing down things I can do to like improve my mental health because I felt like it had already improved’ (Service User 20, 19 years old)*

Clinicians also reported that My Journey 3 was helpful for the service users whom they were supporting.

*“He reported it being useful and because especially with his circumstances he’s really chronically unwell, and there hasn’t been all that much over the past year that’s made a difference to his mental state, so if this is something that he reports being reasonably helpful then that’s a big difference really. Significant.” (Clinician 3)*

However, the age of service users was reported by clinicians as a factor that may impact engagement with My Journey 3 and overall effectiveness. Younger service users were perceived as more likely to use My Journey 3 in comparison to their older counterparts:

*“ … I think especially for younger people perhaps, ‘cos they’re more into apps and Smartphones and what have you, so perhaps that demographic is more likely to use the app, you know … and maybe some of the older participants might not be as keen on apps and Smartphones” (Clinician 9)*

### Burden

Clinicians commonly reported that My Journey 3 potentially created extra work for them. Due to workload pressures, they were often unable to provide as much support to service users as intended, with My Journey 3 sometimes not seen as a priority in their role.

*“I think just on top of everything that I have to do, I felt that the app was kind of pushed to the back …” (Clinician 6)*

*“I guess by January it wasn’t fresh in my mind and during visits it didn’t occur to me to revisit it. But if there weren’t kind of more pressing things to deal with and if it had been in my mind, I would have liked to have had a look at it with him.” (Clinician 8)*

One clinician mentioned that completing the Relapse Prevention Plan and My Recovery Plan on My Journey 3 with a service user would duplicate work they had done previously, and that it was not feasible to repeat that work.

*“I have, in Rio [patient record database], I haven’t done that with the app, I wouldn’t really look to duplicate work in that way because it’s not practical”. (Clinician 3)*

On the contrary, another clinician found that the addition of My Journey 3 to their role was useful and facilitated recovery work with a service user.

*“The fact that we were doing the wellbeing plan and relapse prevention work at the same time she was using the app was really helpful ‘cos it completely suited it.” (Clinician 2)*

According to clinicians, the app relieves service users from going over a *“stack of paperwork” (Clinician 13, Community Psychiatric Nurse)* by providing a digital version of their recovery plan which is also easily accessible.

*“I use my phone more than I look at paper, yeah like I‘d be more inclined to sort of look at the notes that I‘ve created with my care coordinator on my phone” (Service User 21, 35 years old)*

Although only a few service user participants reported that aspects of My Journey 3 were a burden, a recurring theme was the repetitiveness and length of the symptom and early warning signs tracker. This in turn impacted how often service users would use My Journey 3.

*“My main gripe was the fact of having to go through 18 questions every time you track your mood, so because of that it’s not something that I would do every day.” (Service User 7, 27 years old)*

### Opportunity costs

Participants’ views on any benefits and values that they had to give up to engage with the intervention, or deliver the intervention were explored. A few participants were reluctant to save personal information on My Journey 3 due to a possible risk of that information being exposed.

*“I’m paranoid about putting my information in, if I put all my personal information on a phone, you’ve got ways of losing your phone and things like that so I mean on my phone, if you check my phone, I’ve got nothing personal on my phone whatsoever and I didn’t want to store something on an app” (Service User 2, 50 years old)*

A small number of clinicians stated that they would not sacrifice time in clinical appointments if the app was not central to the user at that time point and that they would focus on other priorities instead.

*“I suppose whenever I see him it’s not central to me seeing him, so there are other things that we discuss, and I suppose he’s got other priorities” (Clinician 5)*

### Intervention coherence

Most service users had a clear understanding of what My Journey 3 was, and its purpose. A common theme that emerged was the centrality of the training session in providing an understanding of My Journey 3.

*“I think people need to be shown how to use it which you have” (Service User 19, 22 years old)*

*“Just ‘cos it [the training session] broke down every section of the app, and it was jointly with the client, so you both had the same information so when you came back to discuss it again you could refer back to that meeting and talk about it” (Clinician 2)*

A few clinicians, however, did not seem to fully understand how they could support service users with My Journey 3, other than providing technical support. This is despite being encouraged to actively support the completion of specific recovery and relapse prevention planning sections.

*“ … I don’t really know how else I could support him other than just helping him access it in time when he can’t” (Clinician 3)*

### Self-efficacy

Confidence levels with using My Journey 3 varied among service users and clinicians. Some service users felt the app was straightforward and did not require additional help or support from EIP service professionals.

*“I mean for me it was pretty easy to use so I don’t know what the additional support would have added necessarily” (Service User 7, 27 years old)*

However, other service users felt they lacked support and did not have the confidence to use the various features of My Journey 3. For example, one service user reported that they received little input from their supporting clinician with My Journey 3, and as a result felt that they did not fully use the app as intended.

*“I think perhaps it would be better if I had someone who would be working with me with it, so it wasn’t necessarily used to its full capability” (Service User 12, 27 years old)*

Furthermore some participants found aspects of My Journey 3 complicated, which had a negative impact on how often they used it.

*“Well I felt, actually using the other parts of the app were kind of hard, like I haven’t got to using those parts yet, but the only part of the app that I’ve actually used is the part where it asks you questions and you click yes or no [the symptom tracker].” (Service user 15, 20 years old)*

Severity of service users’ psychotic symptoms was reported to impact the ability to use the app. Experiences such as delusions and paranoia were highlighted as a barrier to using My Journey 3 by service users.

*“If I was really ill, I don’t think I’d be able to use the app because of my fear of surveillance and phones” (Service User 12, 27 years old)*

Clinicians’ confidence in supporting service users with My Journey 3 was mixed. A number of clinicians detailed how they had worked with service users to incorporate My Journey 3 in consultations, and in general reported that they felt well-equipped to provide support.

*“Well, no I do feel confident in providing him with support … he hasn’t raised any as I said problems with it, and I guess if he did then I would be proactive in helping him with it, but at the moment he’s just doing his thing with it. But yeah I’m quite confident to support him with it” (Clinician 9)*

On the other hand, some clinicians expressed a need to be more familiar with My Journey 3 in order to provide the best possible support.

*“I’d say it would probably be good to download the app and if it was used more frequently … so more familiarity with the app would mean I was more likely to access it, information from it and that I’d be better placed to help a client who’s using it” (Clinician 3)*

### Ethicality

Only a few participants discussed the alignment between My Journey 3 and their values regarding giving or receiving care. Clinicians were more inclined to offer support around My Journey 3 to service users if they had positive attitudes towards technology in general. For example, one clinician offered only minimal support because of a general lack of an understanding and interest in Smartphones and apps:

*“I don’t understand, I’m too old, I don’t understand apps, I don’t understand a load of stuff that goes on on Smartphones and I kind of keep it at arms’ length somewhat because it phases me” (Clinician 12)*

One clinician expressed the fear of turning the focus on technology rather than the service user, even though the tool was designed to complement rather than replace face to face contact:

*“ … the danger is that the attention goes on the app rather than on the person and it should be there in the background but a useful barometer and not the be all and end all” (Clinician 5)*

### Suggestions for improvement

A significant theme was that an adapted My Journey 3 could be a good source of social support that would facilitate recovery. Service user participants believed that they would benefit from being able to send messages and information to their clinician or friends and family members through the app. A number of participants said they would benefit from having access via My Journey 3 to a forum where they could interact with peers who have experience of psychosis.

A senior practitioner suggested that My Journey 3 could provide a digital version of an advance statement that sets down the service user’s values, beliefs, and wishes regarding their future care in case they become unwell and lose decision-making capacity.

## Discussion

The aim of this study was to explore EIP service user and clinician perceptions on the acceptability of a supported self-management Smartphone app (My Journey 3). Although responses were specific to My Journey 3, much of the feedback is likely to be relevant to similar DHIs delivered within EIP services. In analysing and reporting our data, we were especially interested in understanding the relatively low rates of app usage found for My Journey 3 [15], which are characteristics of the use of many mental health apps in real world settings and a potential challenge to unlocking the potential of apps in clinical practice.

Many service user participants felt that My Journey 3 was acceptable and reasonably simple to use. Functions in My Journey 3, such as the symptom tracker and activity reminders, were reported as useful and several users attributed positive changes in their lifestyle, routine, and medication adherence to the DHI, providing promise that My Journey 3 could be an effective healthcare tool. Furthermore, service user participants reported that My Journey 3 encouraged them to reflect on their experiences and improved their control and involvement in their mental health care. This echoes previous findings where EIP service users reported that DHIs have the potential to increase choice, control and empowerment [4].

Two key concerns regarding acceptability for service users were apparent. First, self-efficacy in relation to engaging effectively with My Journey 3 was reported by some participants to be low, hampering use even though all participants reported being active technology users and found the training session beneficial. People with psychosis have previously been reported to have reservations about whether they have the necessary skills and experience to engage effectively with DHIs [26, 27]. Means to simplify content in collaboration with service users and providing more targeted support with My Journey 3 should be considered to limit non-engagement and potential digital exclusion [28]. Such strategies are likely to be of particular importance in the early stages of new technology use in order to embed long-term engagement [29].

Second, a number of service users held concerns regarding data protection, privacy, and security of information, an inherent issue with DHIs. These concerns were more apparent for some services users than others, and perhaps were a key factor in the wide variability of engagement with My Journey 3 within the ARIES study [15]. Recent qualitative literature suggests that this a common concern for individuals with psychosis [4, 27], with clearer privacy policies and greater reassurance of users’ data security two possible measures to allay concerns and increase acceptability.

Service users’ reports suggested some relationship between interest in using My Journey 3 and current stage of recovery and symptoms. This is in keeping with other accounts, for example from an expert consensus survey that suggested severe positive symptoms are likely to reduce user engagement with DHIs [30]. Similarly more severe negative symptoms have been shown to impact internet use for adults with psychosis [31], and could perhaps influence engagement with other digital activities including Smartphone apps. User engagement may increase if DHIs can be suitably tailored and adapted to the users’ level of recovery to ensure content is relevant [32]. DHIs, including My Journey 3, need to be suitable and acceptable for those with higher symptom severity in order to avoid digitally excluding those who may benefit most [33]. Older age was also suggested as a factor that could also impact service user engagement and clinician adoption of the app, in line with evidence that younger adults have longer continued engagement with DHIs [28].

The majority of suggestions to improve My Journey 3 focused on how it could foster and increase communication with peers and clinicians. Building social support is a key strategy in illness self-management for psychosis [34], contact with peers with lived experience is an important facilitator of recovery [35] and peer-support is recommended in current NICE guidelines in the UK [36]. Greater peer-to-peer communication could potentially be incorporated in the app, with care taken to prevent issues regarding confidentiality and data protection.

Based on service users’ feedback symptom and medication tracking along with activity reminders were viewed as the most beneficial aspects. Accordingly, the simpler independent aspects of self-management may be more readily accepted and engaged with in DHI format that more complex sections benefiting from clinician involvement, such as crisis and relapse prevention plans. Feedback suggested that these sections were harder to use, while the symptom tracker was the most used section in the ARIES study [15]. Symptom monitoring has been found in other studies [4, 16] to be valuable and acceptable for people with psychosis, and to help facilitate shared decision making [37]. However, the primary aim of the ARIES study was to support and improve implementation of complex self-management tasks, such as crisis planning and relapse prevention, which our findings suggest is more challenging.

While clinicians expressed broadly positive views about My Journey 3, many of them did not prioritise supporting service users with their use of it over clinical priorities seen as more pressing or relevant to achieving mandated service targets. Additional workload has previously been cited as a key implementation barrier [38, 39], with the “burden” of app-based work a prevalent construct in clinician interviews. Digital tools are likely to be more acceptable to clinicians if they are not time-consuming and are integrated with current care systems so that clinicians derive clear benefits in their work from engaging with them. Data-sharing between apps and healthcare records was not part of our study and has been argued to be essential if clinicians are to be highly engaged with DHIs [2]. Our findings cohere with qualitative findings from the development phase of the Actissist and EMPOWER apps, where it was also reported that clinicians in EIP settings approve of DHIs, but are much more likely to engage with them if they help them achieve existing targets and complete required processes, and if practical and organisational support are provided [40, 41]. To note, one of the most successful DHIs delivered in psychosis care, Virtual Reality therapy, is delivered by a designated therapist allowing far greater involvement and support [42]. One way to effectively implement DHIs within mental health services, and to overcome staff “burden” may be through designated digital “champions” whose specific role is to support and assist with DHI training and integration [43].

There was some suggestion that acceptability was linked to clinicians’ confidence and familiarity with the app and technology in general, in line with past research [37], and accordingly training should be tailored to this. Although not mentioned in the interviews, clinician training was not manualised and did not cover potentially significant areas such as trouble-shooting technical issues, and effective integration within clinical care which could have impacted clinicians’ self-efficacy and consequently service users’ level of engagement.

### Implications

We were able to find both service users and clinicians who were interested and willing to try out a self-management app for psychosis, and their reports suggested that on most dimensions it was acceptable. However, we found shortfalls in acceptability and in support for implementation that offered explanations for the fact that usage of the app was not sustained over months by most, despite initial enthusiasm [15]. Our findings suggested that service users would be more likely to sustain app use if it were more integrated into their care and supported by clinicians, and that clinicians would be more likely to prioritise it despite time burdens if it were more obviously helpful in doing their job effectively and efficiently, more clearly supported by and embedded in their organisation, and more relevant to the targets that they are expected to meet. A recent meta-analysis has suggested that Smartphone apps used across a range of mental health problems are not effective when used independently [44] further suggesting that developing and testing apps that are more integrated with clinical care and for which clinician support is more readily mobilised may be an appropriate direction for further DHI development and testing. Authors of a recent scoping review suggest that when DHIs are used collaboratively by service users and clinicians they can enhance positive relationships that are central to recovery-oriented practice [45]. Thus, in future research that evaluates the effectiveness of self-management DHIs in promoting recovery in EIP services, implementation science theories and frameworks should be used to examine how to overcome delivery barriers that prevent effective integration into clinical practice.

Prior to further evaluation of My Journey 3, acceptability and sustained use need to be improved through further refinement of the intervention and its delivery. Our findings suggest potential strategies to increase service user engagement, including modifications to the app to simplify complex tasks such as relapse prevention planning as much as possible, and better technical support and reassurance regarding privacy and security. At clinician- and service-levels, integration with local electronic systems and assessment and care planning processes, support from managers at all levels, and digital champions or staff with dedicated time to deliver the intervention are among the potential strategies to increase sustained use. A blended approach, with service users and clinicians choosing between face-to-face and digital self-management tools that can be customised to individual needs appears to fit with service user preferences. Involvement of both service users and clinicians in developing co-produced ways of addressing implementation barriers is also likely to be key [19]. Furthermore the addition of peer support as part of a DHI for first-episode psychosis appears important for user experience and engagement and is currently being explored [17] in a RCT of a peer-supported early warning signs monitoring app (EMPOWER).

### Strengths and limitations

Our study is one of the first to obtain the views of both EIP service users and clinicians on a self-management Smartphone app. Gaining the perspectives of both the deliverers and recipients of healthcare interventions has been highlighted as important for successful implementation of an intervention [19], and in this study these perspectives cohere in allowing us to identify significant implementation barriers for this technology. A diverse sample of both EIP clinicians and service users was recruited, including almost all those participating in the wider research project.

Our findings need to be considered in light of the study limitations. All service user participants owned an Android Smartphone and were recruited from a larger research study investigating My Journey 3. As a result, our study may have involved service users who were already open to and interested in digital technologies, and have an existing understanding of Smartphones and apps, however only a small number had used mental health apps previously. The recruitment process is likely to have favoured service users with a positive view of technology [46]. The generalisability of our sample is further limited by excluding non-Android Smartphone owners.

Furthermore, responses could be influenced by social desirability bias. Service user and clinician participants were already familiar with the interviewer from previous research meetings and may have felt inclined to provide positive feedback or exaggerate the level of app use or the amount of support given, despite being encouraged to provide honest feedback. The researchers conducting the interviews were neither qualified clinicians nor service users, potentially also limiting disclosure.

Finally, at the time of interviews service user participants had had access to My Journey 3 for approximately 7 weeks on average. Enthusiasm and engagement with Smartphone apps is likely to be highest in the initial few weeks of use, with results from our trial showing that 50% of participants disengaged with My Journey 3 within the first 3 months of use [15]. As such our findings may capture users’ opinions of My Journey 3 at the time when they were most engaged with it, meaning feedback is likely to be more positive than if interviews were conducted at a later time-point.

## Conclusions

The study reports both EIP service user and clinician feedback on a supported self-management Smartphone app. Many EIP service users successfully engaged with the intervention and reported benefits to their medication adherence and lifestyle and felt an increased feeling of control over their illness. Clinicians placed a significant emphasis on the low level of support they were able to provide due to other workload pressures. These findings contribute to the growing evidence that DHIs can be used to support self-management for adults with psychosis and can further inform the development of My Journey 3 and its potential delivery in EIP services.

## Data Availability

The datasets generated during and/or analysed during the current study will be made available two years after the study end.

## Abbreviations

EIP: Early Intervention in Psychosis
DHI: Digital health intervention
NHS: National Health Service
ARIES: App to support Recovery In Early Intervention Services
RCT: Randomised controlled trial
